# Different Types of Chronic Inflammation Engender Distinctive Immunosenescent Profiles in Affected Patients

**DOI:** 10.3390/ijms232314688

**Published:** 2022-11-24

**Authors:** Eleni Moysidou, Georgios Lioulios, Aliki Xochelli, Vasiliki Nikolaidou, Michalis Christodoulou, Zoi Mitsoglou, Stamatia Stai, Asimina Fylaktou, Aikaterini Papagianni, Maria Stangou

**Affiliations:** 1 School of Medicine, Aristotle University of Thessaloniki, 45642 Thesaloniki, Greece; 2 Department of Nephrology, Hippokration Hospital, 54642 Thessaloniki, Greece; 3 Department of Immunology, National Histocompatibility Center, Hippokration General Hospital, 54642 Thessaloniki, Greece

**Keywords:** end-stage kidney disease, systemic lupus erythematosus, lymphocytes, naïve cells, memory cells, senescence

## Abstract

Immunosenescence encompasses a spectrum of lymphocyte phenotypic alterations. The aim of the study was to evaluate immunosenescent effect of two different forms of chronic inflammation, Systemic Lupus Erythematosous (SLE), a systemic autoimmune disease, and End-Stage Kidney Disease (ESKD), a chronic inflammatory disorder. Certain lymphocyte surface molecules, including CD31, CD45RA, CCR7, CD28, CD57, for T, and IgD, CD27 for B lymphocytes, were analyzed by flow cytometry in 30 SLE and 53 ESKD patients on hemodialysis (HD), and results were compared to 31 healthy controls (HC) of similar age, gender, and nationality. Significant Lymphopenia was evident in both SLE and ESKD-HD patients, compared to HC, affecting B cells 75.4 (14.4–520.8), 97 (32–341), and 214 (84–576) cells/μL, respectively, *p* < 0.0001, and CD4 cells 651.2 (71.1–1478.2), 713 (234–1509), and 986 (344–1591) cells/μL, respectively, *p* < 0.0001. The allocation of B cell subpopulations was remarkably different between SLE and ESKD-HD patients. SLE showed a clear shift to senescence (CD19IgD-CD27−) cells, compared to ESKD-HD and HC, 11.75 (10)% vs. 8 (6) vs. 8.1 (10), respectively. Regarding T lymphocytes, Central Memory CD8 cells predominated in both SLE and ESKD-HD patients compared to HC, 53 (50)%, 52 (63), and 24 (64)%, respectively, while ESKD-HD but not SLE patients also had increased expression of CD4CD28− and CD8CD28− cells. In conclusion, both diseases are followed by significant lymphopenia; however, the senescent phenomenon affects the B lymphocyte compartment in SLE patients and T lymphocytes in ESKD-HD patients.

## 1. Introduction

In the recent years, increasing research into the phenomenon of immunosenescence has demonstrated its clear association with inflammation. Three distinct characteristics signify the process of immunosenescence, that is, involution of thymus, leading to a reduction of naïve T lymphocyte population, followed by a shift towards advanced differentiated subtypes, contraction of T cell repertoire with oligoclonal expansion of naïve and memory/effector T cells demonstrating a replicative insufficiency after repetitive activation, and a chronic inflammatory status called inflammaging [1,2,3], a phenomenon also present in autoimmune diseases such as rheumatoid arthritis [4].

Phenotypic alterations of T lymphocytes, secondary to aging, include downregulation of CD31, representative of Recent Thymic Emigrant (RTE) cells, CD27 and CD28 molecules, and upregulation of CD57 and KLRG1, as well as the co-expression of TIGIT and Helios molecules, and the re-expression of CD45RA molecule in effector memory cells, resulting to CD45RA+CCR7− cells, defined as T effector memory cells re-expressing CD45RA (TEMRA) [5,6]. Chronic inflammation is proved to cause substantial functional and phenotypic alterations to the innate and adaptive immune system, similar, though not identical, to those observed at the process of aging. However, chronic inflammation is not an ordinary ailment or a coherent disorder; it can derive as a result of systemic immunological diseases, metabolic syndromes, chronic infections, but also, environmental or lifestyle factors. [1,7,8].

End-stage kidney disease (ESKD), a typical condition of persistent inflammation due to uremic environment, oxidative stress, and dialysis method, is characterized as a chronic inflammatory disorder, followed by disturbed immune activity and weakened adaptive immunity, which is reflected by the inadequate response to vaccines and the high incidence of infections and curtain tumors [9,10]. We have previously described the characteristic changes in the immunosenescent profile of patients with ESKD undergoing hemodialysis, including significant lymphopenia, affecting mainly RTE and naïve subpopulations of CD4 cells, reduced expression of CD28 molecule on both CD4 and CD8 cells, and severe B cell lymphopenia, mainly of switched memory cells. The above lymphocyte modifications are similar to aging in some points; however, there are certain differences, probably attributed to distinct mechanisms. [1,11,12,13].

Systemic lupus erythematosus (SLE) is an autoimmune systemic disorder, with an unknown etiology, associated with dysregulation in the interaction between innate and adaptive immunity, breakdown of self-tolerance and production of auto-autoantibodies. Activation of the adaptive immune system by the innate immune system is mainly responsible for the tissue inflammation and damage present in autoimmune diseases, including SLE. There is a partial distinction from autoinflammatory diseases, where the innate immune system comprises a basic causative factor of damage, without the involvement of autoantibodies or autoreactive antigen-specific T lymphocytes. Yet, over the last years increasing evidence highlights the connection between autoimmunity and autoinflammation, emphasizing on the role of innate immunity, certain cytokines, such as IL-1β, and inflammasomes [14,15,16].

Very interestingly, most studies regarding immunosenescence in SLE revealed an upregulation of advanced differentiated lymphocytes, for instance, CD28null-angiogenic T cells, CD4+KLRG1+, CD8+KLRG1+, and CD8+CD57+ cells, with no significant changes in naïve populations.

Therefore, a discrepancy seems to exist between the immunosenescent profile (CD57+, KLRG1+, CD28null) between chronic inflammatory situations, indicating and predicting distinct clinical consequences [17,18,19]. Moreover, it is well known and widely accepted that the presence of ESKD and start on dialysis substantially reduces and finally eliminates SLE activity [20].

Although the effect of both these two chronic inflammatory conditions (SLE and ESKD) on phenotypic characteristics of T and B cell subpopulations has been thoroughly studied over the last few years, demonstrating various features of premature immunosenescence, there are no reports in the literature, addressing a comparison between the two. Therefore, the aim of our study was to describe, evaluate, and compare the impact of SLE and ESKD on the immunosenescent process, in terms of cellular phenotypic changes.

## 2. Results

### 2.1. Characteristics of Patients’ Population

Thirty patients with SLE (29 female) and fifty-three ESKD-HD patients (19 female) were included in the study. The control group consisted of 31 healthy individuals (17 female), mean age of 49 ± 13 years, similar to patients groups, SLE 43 ± 14 years, ESKD-HD 47 ± 14 years, *p* = 0.696.

Mean SCr of SLE patients was 0.89 ± 0.17 mg/dL, eGFR was 81 ± 19 mL/min/1.73 m^2^, Uprot 680 ± 5.600 mg/24 h, and C3 and C4 levels at time of evaluation were 74.65 (29.9–127) mg/dL and 15.7 (3.86–27.2) mg/dL, respectively. Median SLEDAI score was estimated to 10 (2–18) at time of diagnosis and 2 (1–5) at time of evaluation. Twenty SLE patients (67%) had lupus nephritis, based on renal biopsy, which had been performed not more than 5 years prior to evaluation. Median time since initial SLE diagnosis was 84 (45–125) months.

ESKD-HD patients (19 female) had been on HD for a period of 67 (28–84) months. All patients had KT/V > 1.5 and dialyzed for at least 4 h three times per week. Table 1 describes demographic and basic laboratory findings at time of evaluation. All SLE patients were on hydroxychloroquine at time of assessment, 24 (80%) were on steroids, 20 (67%) on MMF, and 12 (40%) on CNIs. Data regarding medication and time on immunosuppression are described on Supplementary Table S1. Twenty of patients (67%) had taken cyclophosphamide in the past, at least 2 years ago, according to inclusion criteria. No one had received monoclonal antibodies.

### 2.2. Phenotypic Analysis of B Lymphocytes in SLE, ESKD-HD Patients and HC

Total WBC of SLE, ESKD-HD patients, and HC was 7200 (3350), 7100 (1950), and 6400 (1800) cells/μL, respectively, *p* = 0.154. Total lymphocytes were significantly reduced in both SLE and ESKD patients compared to HC, 1400 (900), 1500 (500), 2100 (900), respectively, *p* = 0.001 (Table 1).

**Figure 1:**
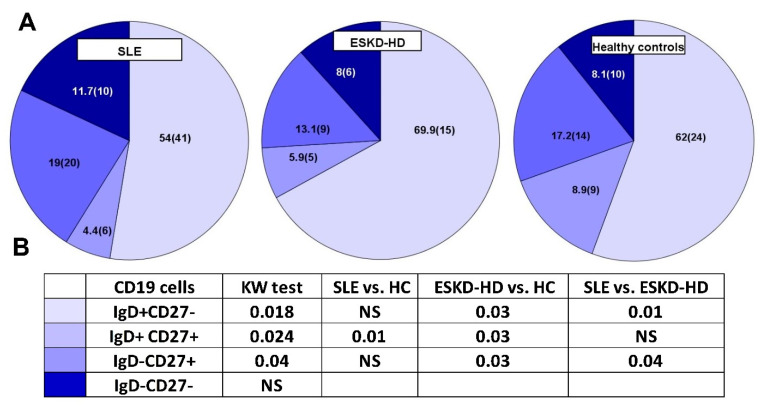
Median percentage (IQR) of B Lymphocyte subpopulations, Naïve (IgD+CD27−), IgM memory (IgD+CD27+), Switched memory (IgD−CD27+), and Late differentiated (IgD−CD27−) (**A**), and demonstration of statistical differences between the three groups, SLE and ESKD-HD patients and Healthy Controls (**B**). (KW test: Kruskal–Wallis test and Dunn test).

The percentage and absolute numbers of B lymphocytes were significantly reduced in both SLE and ESKD patient groups, as compared with the HC group, 6.8 (2.1–28.6)% vs. 7.1 (1.7–21.4)% vs. 11.8 (5.4–24)%, respectively, *p* < 0.001, and 75.4 (14.4–520.8)cells/μL vs. 97 (32–341)cells/μL vs. 214 (84–576)cells/μL, respectively, *p* < 0.001. This reduction affected all subpopulations in both patient groups; however, their distribution was altered in a completely different manner (Table 2 and Figure 1). Interestingly, SLE patients seemed to have the highest percentage of the senescent subtype, IgD-CD27−, compared to both ESRD-HD and HC. More specifically, compared to HC, SLE patients had reduced percentage of naïve, IgD+CD27−, 62 (24)% vs. 54 (41)%, IgM memory, IgD+CD27+ 8.9 (9)% vs. 4.4 (6)%, respectively, and increased percentages of switched memory, IgD-CD27+ 17.2 (14)% vs. 19 (20)% and late differentiated B lymphocytes, IgD-CD27− 8.1 (10)% vs. 11.7 (10)%, respectively. The B lymphocyte compartment of HD patients, on the other hand, consisted of 69.9 (15)% IgD+CD27−, 5.9 (5)% IgD+CD27+, 13.1 (9)% IgD-CD27+, and 8 (6)% IgD-CD27− cells. The median percentage and allocation of B lymphocyte subpopulations are depicted in Figure 1, and their counts in each group are described in Table 2.

**Figure 2:**
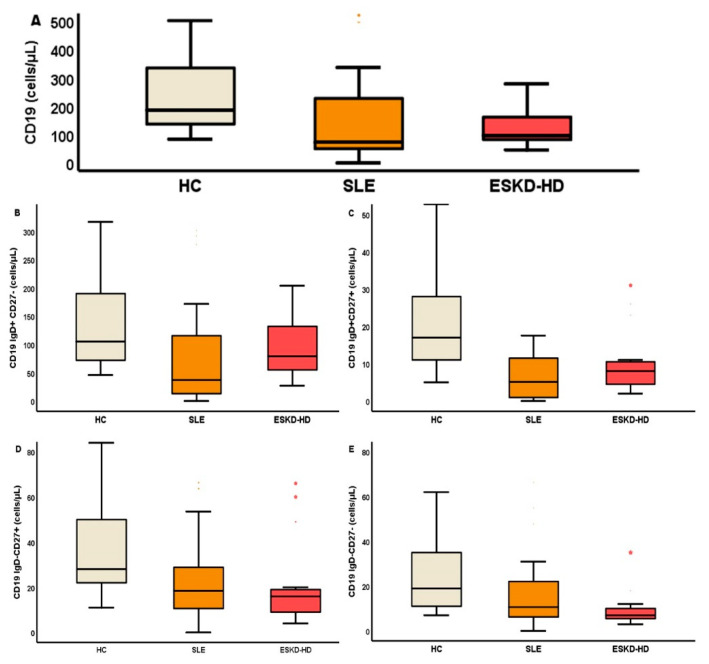
Total number of CD19 lymphocytes (**A**) (cells/μL) and of their subpopulations IgD+CD27− (**B**), IgD+CD27+ (**C**), IgD-CD27− (**D**), and IgD-CD27− (**E**) (cells/μL), represented by box plots.

The most important differences of B lymphocyte subpopulation counts are depicted in Figure 2. The statistical differences between the three groups and post hoc analysis are described on Table 2. As there was a significant reduction of B lymphocytes in both SLE and ESKD patients compared to HC, we decided to estimate the ratio of naïve over late differentiated subtypes in all groups to further estimate the modification of cell preponderance. The ratio of CD19IgD+CD27−/CD19IgD-CD27− count was 7.6 (7), 4.6 (6.2), and 9.5 (9), respectively, *p* = 0.01.

As the vast majority of SLE patients were female, we decided to make all statistics in females only, and results are described in the supplement file (Supplementary Table S2). Interestingly, the above predominance of senescent subpopulations in SLE patients was even more prominent in female patients and differences of the CD19IgD-CD27- cell count between SLE, ESKD-HD patients, and HC were significant, 10.64 (0.93–122.91), 7 (3–35), 19 (7–62) cells/μL, respectively, *p* = 0.003.

### 2.3. Phenotypic Analysis of T Lymphocytes in SLE, ESKD-HD Patients and HC

#### 2.3.1. CD4 Lymphocytes

**Figure 3:**
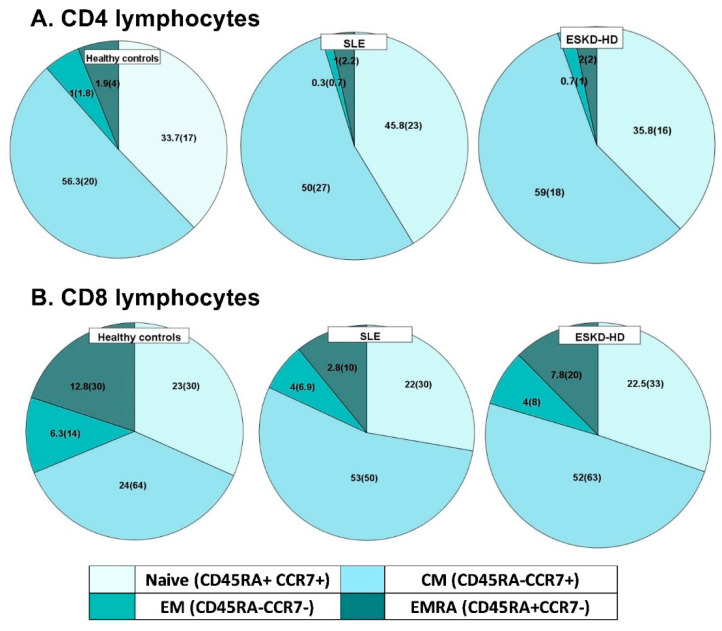
Percentage of CD4 (**A**) and CD8 (**B**) Lymphocyte subtypes.

Total CD4+ lymphocytes were significantly reduced in both SLE and ESKD-HD patients, compared to healthy controls (651.2 (71.1–1478.2) cells/μL, 713 (234–1509) cells/μL and 986 (344–1591) cells/μL, respectively, *p* < 0.0001). Their percentages expressed as Median (IR) are depicted in Figure 3, which illustrates differences in the allocation of T lymphocytes between the three groups. Panel A depicts differences of the CD4 subpopulations, showing the reduction especially of EM and EMRA CD4 subtypes, in SLE patients, compared to HC.

**Figure 4:**
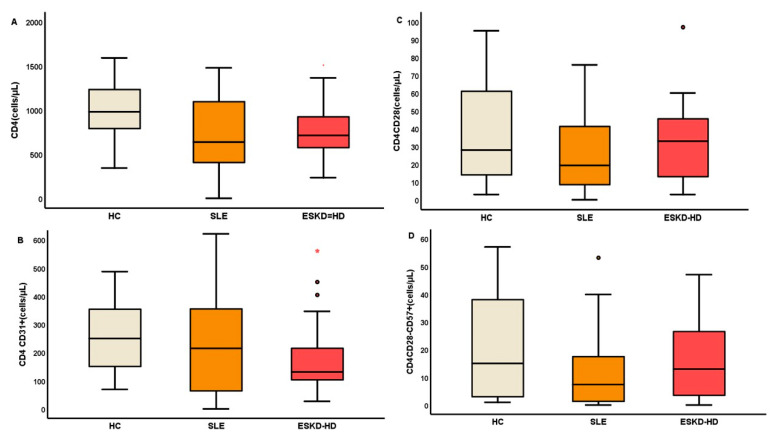
The population of total CD4 cells (**A**), CD4CD31+ (**B**), CD4CD28− (**C**), and CD4CD28-CD57+ (**D**) (cells/μL) are shown as the most important of early and late differentiated cell subtypes. (SLE: Systemic Lupus Erythematosus, ESKD-HD: End-Stage Kidney Disease on Hemodialysis).

The count of CM (CD4CD45RA-CD57+), EM (CD4CD45RA-CD57−), and EMRA (CD4CD45RA+CD57−) CD4+ subpopulations were significantly reduced in SLE patients compared to the healthy control group; *p* = 0.046, *p* = 0.002, *p* = 0.027, respectively. Interestingly, there were no remarkable changes in the rest of senescent CD4 subtypes, as there were defined by the presence or absence of CD57 and CD28 surface molecules; Table 3. The main and most important differences in CD4 subtypes are shown in Figure 4.

As for the ESKD-HD population, there was a significant reduction in naïve (RTE and CD4CD45RA+CCR7+) (Table 3) and early differentiated subtypes (CD4CD45RA+CD28+, CD4CD28+CD57−, CD4CD45RA+CD57−, CD4CD45RA-CD57−), compared to healthy controls; however, no differences were found in the allocation of the different CD4+ subtypes (naïve 35.8 (16)% vs. 33.7 (17)%, CM 59 (18)% vs. 56.3 (20)%, EM 0.7 (1)% vs. 1 (1.8)%, EMRA 2 (2)% vs. 1.9 (4)%, respectively; Figure 3.

The statistics in CD4 cells and subtypes are described in the supplement file (Supplementary Table S3).

#### 2.3.2. CD8 Lymphocytes

CD8+ lymphocytes demonstrated a trend towards lower absolute counts in both groups of patients (Table 4), while changes of subtype proportion were overall less distinct among the under-study individuals. However, the analysis interestingly showed a statistically significant elevation of the percentage of CM cells in SLE, 53 (50)% and ESKD-HD patients, 52 (63)%, compared to HC, 24 (64)%. Indeed, in SLE, the redistribution also affected the EMRA subpopulation, which displayed a significant reduction compared to HC. Noteworthy, in the same patients, apart from the EMRA cells, there was an obvious reduction in two senescent subpopulations, named CD8+CD45RA+CD28− and CD8+CD45+CD57+ cells, compared to HC (Table 4).

**Figure 5:**
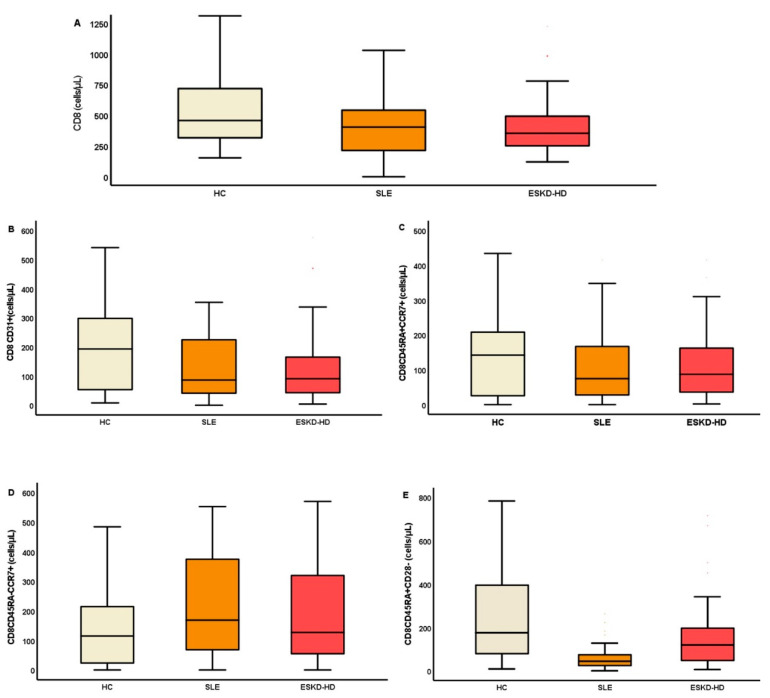
The population of total CD8 cells (**A**), CD8CD31+ (**B**), CD8CD45RA+CCR7+ (**C**), CD8CD45RA-CCR7+ (**D**), and CD8CD45RA+CD28− (**E**) (cells/μL) are shown as the most important of early and late differentiated CD8 cell subtypes.

On the contrary, ESKD-HD patients showed a more immunosenescent phenotype in terms of upregulation of CD57 together with elimination of CD28 molecule. Namely, the reduction in ESKD-HD patients affected naïve/middle aged cells (CD8+CD31+, CD8+CD45RA+CD28+, CD8+CD28+CD57−, CD8+CD45RA+CD57−) and is describe at Table 4. Most important differences are shown in Figure 5, by box plots representing the population of lymphocyte subtypes by box plots (cells/μL).

Statistics performed in females only showed a similar re-distribution of CD8 cells, with a reduced expression of naïve and increased central memory CD8 cells in both SLE and ESKD-HD patients.

The results regarding changes in CD8 cells and their subtypes are described in the supplement file (Supplementary Table S4)

### 2.4. Adjustment of Lymphocyte Subpopulations for Age and Gender

Based on the effect of ageing in lymphocyte senescent markers, we decided to estimate the impact of age in our results. Therefore, correlation of age with specific B and T lymphocyte subpopulations have been performed and presented at heatmap Tables S5 and S6, respectively.

Moreover, as there was an obvious unbalance in our population in terms of gender, with females predominating in the SLE group, we performed the same statistics separately for females only. We also performed type II ANOVA to adjust the effect of age and gender in lymphocyte subtypes, and type III ANOVA to estimate the interaction of these parameters. Age had significant negative correlation with total CD19 cell count, and also naïve, CD19IgD+CD27− cells and late differentiated B cells, CD19IgD-CD27− cells. Although differences in B lymphocyte subpopulations are remarkable and significant between different groups of patients, the effect of age is also important, as seen in Supplementary Tables S5 and S7 and Supplementary Figure S3.

Regarding T lymphocytes, Supplementary Tables S6 and S7 and Supplementary Figures S4 and S5 demonstrate the negative correlation between age and naïve CD4 and CD8 sub-lymphocytes and also a significant positive correlation between age and senescent CD8 subtypes.

Compared to the results from Kruskal–Wallis H test, when we performed ANOVA to correct results for age and gender, we noticed that several CD4 subpopulations had a significant impact from age. CD4CD45RA+CCR7+, total CD8, CD8CD45RA+CD28−, CD8CD45RA+CD57−, and CD8CD45RA+CCR7− cells seemed to retain their significant alterations after adjustment for age. Senescent cell populations, CD4CD45RA+CCR7−, CD8CD45RA+CCR7−, CD8CD45RA+CD28− were still different between disease groups, regardless of the impact of age.

## 3. Discussion

In the present study, we investigated the effects of two different forms of chronic inflammation on the immune cell phenotype and process of lymphocyte activation and maturation. The specific results of SLE, as a systemic autoimmune disease, and of ESKD, as a systemic chronic inflammation, on the progress of lymphocyte differentiation, were investigated and compared with that of healthy individuals. To our knowledge, this is the first study which compares phenotypic alterations of B and T lymphocytes in two forms of systemic chronic inflammation. We have previously described the actual and unique consequences of chronic kidney disease on lymphocytes’ structure and function, resembling that of ageing, however, retaining substantial differences [11,12,13]. The detrimental effects of renal impairment on the immune function are attributed to uremia, uremic toxins, dialysis method per se, oxidative stress, and probably to other yet unrecognized causes [1]. It would be interesting to investigate whether the devastating results on the immune system, regarding the maturation and senescent progress, are similar or divergent in two chronic and sustained situations. Therefore, we evaluated the immunosenescent profile of B and T lymphocytes in patients with SLE and those with ESKD-HD and healthy population.

A significant reduction in the total B lymphocyte numbers was noticed in both SLE and ESKD-HD patients, compared to healthy controls, with this reduction being even more prominent and important in SLE patients. B cell lymphopenia is well known and has been extensively described in both diseases; however, the most interesting point in our study was the specific effect on the individual B cell subsets, which was completely different in SLE compared to ESΚD-HD patients. In SLE patients, a sharp drop-off in naive and early differentiated subtypes (IgD+CD27− and IgD+CD27+) was remarkable, with a clear shift to more senescent forms (IgD-CD27−). ESΚD-HD patients, on the other hand, showed an unexpected increase in the naïve subpopulation, followed by a prompt reduction in memory B cells. As our SLE patients were mainly females, we performed the same statistics in the three groups of patients but including the females only. Very interestingly, differences in the female groups were even more projecting, mainly because women in the ESKD-HD group could better preserve B cells from shifting to senescent phenotype. In addition to this analysis, including females only, we performed a further adjustment for gender to estimate specific effects on our results. Additionally, although the groups of patients and healthy controls were of similar age, we could not exclude the effect of age in all these senescent indices of lymphocytes. Therefore, additional statistics were performed for all lymphocyte subpopulations, which revealed that the above described differences in B lymphocytes and their subpopulations between SLE and ESKD-HD patients remained after adjustment for age and gender.

The specific alterations in SLE are similar to those previously described from other investigators [21,22,23], which showed a combined reduction in the expression of CD27 and IgD, and increase in memory B lymphocytes and plasma cells. The pathogenesis of B lymphocyte changes leading to the specific pattern characterizing SLE, is still unclear. The upregulation of memory B cells and plasma cells could be attributed to the enhanced activation and differentiation of naive B cells, or, alternatively, may be the result of an enhanced breakdown of self-tolerance or a disruption to the processes of selection [24,25]. Meanwhile, in ESΚD the B-cell lymphopenia is proposed to be associated with increased apoptosis and resistance of transitional B cells to vital signaling pathways, such as differentiation and survival, due to the uremic environment [26,27,28].

In spite of the straightforward shift to senescent profile of B lymphocytes in SLE patients, our findings regarding CD4 and CD8 subpopulations, were more complex and probably difficult to interpret, yet revealed some interesting findings. CD4 lymphocytes were profoundly reduced in both SLE and ESKD-HD individuals, nevertheless, quite surprisingly, down-regulation in SLE patients affected mainly advanced differentiated cell types, that is CD4 EM and EMRA cells, as well as CD4CD28− cells, either with or without CD57 expression. Early differentiated forms (naïve and CM) as well as recent thymic emigrants (RTEs) were preserved. This clear shift to less senescent CD4 subtypes in our SLE patients was totally distinct from the pattern seen in ESKD-HD patients, where naïve and RTE cells were reduced, leading to a preponderance of senescent subtypes, especially CD4CD28− cells. The same distribution was preserved when we estimated differences including the female population only. Very interestingly, when results were adjusted for age and gender, some of the above subpopulations, namely, all the early differentiated CD4 subtypes, seemed to retain their important divergence between groups of patients, although a few more senescent populations, CD4CD45RA-CCR7− and CD4CD28− subtypes, lost their significance, indicating that they may be influenced by patients’ age.

Our findings regarding the SLE patients are slightly different from previous studies [29,30,31,32], which demonstrated expansion of the effector and senescent CD4+ T- cell subpopulations in SLE patients compared to healthy controls; nevertheless, they emphasize their superiority mainly in patients with active disease, or those with metabolic syndrome. In our study, all SLE patients were in remission at the time of evaluation. It seems possible that SLE per se, as a low-grade inflammatory condition caused by constant antigenic stimulation, through an over-activation of the immune system, leads to proliferation and differentiation of naïve T cells into activated cell types altering the expression of certain CD4 receptors and leading to senescent subtypes that amplify autoimmunity via the production of pro-inflammatory cytokines during activation [19,33,34], although this effect is potentially reversible when disease goes into remission.

In our ESΚD-HD patients, a remarkable reduction of CD4 cells was clearly manifested, affecting primarily naïve CD4+ cells and RTEs, but with no significant changes in the ratio of subpopulations. This distribution comes to an agreement with previous research, which attributed this shift towards a memory profile, to insufficient thymic function, impaired circulating levels of the IL-7 cytokine, uremic conditions and increased turnover or differentiation to memory T lymphocytes [13,21,35,36,37,38,39,40]. On the other hand, the also moderately reduced numbers of CD8 lymphocytes seem to display a complete redistribution between naïve/CM/EM/EMRA cell types, in favor of CD8 CM cells. This pattern appears to dominate in both SLE and ESKD-HD patients, with similar proportions of subpopulations, as compared to healthy controls. Our results, regarding increased CM cells in SLE are in accordance with previous ones showing accumulation of certain T lymphocyte subsets in active SLE. The predominance of CD8+CD45RO+CCR7+ cells has been related with SLE activity, while their population follows a decreasing course during disease remission [41]. Yet still, conflicting results have been reported by other studies in SLE, presenting a tendency for more differentiated forms, such as EMRA cells, to prevail [29,30,31], characterized by a low susceptibility towards apoptosis [42] and related with SLE activity. It is still unclear how a T lymphocyte is chosen to become a central (CM, CD45RA+, CCR7+) or an effector memory cell (EM, CD45RA−, CCR7−) [33,43] although it seems that apart from antigenic stimulation, the plenty homeostatic cytokines affect differently the differentiation pathway of T lymphocytes [34]. Equally blurred is the scenery in ESKD, as few teams have demonstrated increased proportion of late differentiated effector memory cells (TEMRA) [21,36,37,38] while others [44] doubt their absolute reduction and raise the question of whether the described decline of EM is due to migration to peripheral tissues.

Based on the presence of CD45RA and CCR7, our results showed a preponderance of naïve or central memory cells in SLE compared to healthy controls, while in ESKD patients, the main finding was the down-regulation of naïve populations. In order to investigate these findings further, we evaluated the expression of two molecules, CD28 and CD57, strongly connected to the process of senescence. Repetitive activation of T lymphocytes, in the presence of chronic inflammation and results in the gradual reduction leading to elimination of CD28 receptor, CD57 molecule is gradually expressed in mature T cell subtypes. [1,11,12,13].

Noteworthy was a disturbed fluctuation in CD4 cells, with an evident turn to senescent types, in the peripheral blood of ESKD-HD patients, while SLE patients retained cell proportions similar to that of healthy controls, with a downward trend of the absolute count of all subpopulations, significantly of CD28+CD57-. On the other hand, the senescent phenotype of CD8+ cells was obviously and undoubtedly demonstrated only in ESKD-HD, while SLE patients were followed by rather reduced expression of senescent subtypes, such as CD8+CD45RA+CD28− and CD8+CD45RA+CD57+ cells. More extensive statistics, taking into account patients’ age and gender, confirmed the above alterations in CD8 subpopulations and emphasized the predominance of CD8 senescent phenotype in ESKD-HD patients, although it raised few concerns regarding CD4 cells, indicating that ageing plays a crucial role in the development CD4 senescent phenotype. Previous research over the above described receptors appears to be controversial. More specifically, while many researchers found no difference [30,45] or even significantly lower numbers of CD8CD28null cells [37], others reported an up-regulation, notably connected to SLE activity [46,47,48]. Senescent phenotype of CD4+ cells seems to be more established, as numerous studies in the past have pointed the profound expansion of the CD4CD28null and its association with disease damage and activity [29,30,45,48], not depicted in our analysis, as we included patients on remission only.

In the present study, we focused on the evaluation of lymphocyte surface molecules which highly represent activity and senescence. Apart from the specific cell subtypes, as defined by the certain molecules studied, novel subpopulations are being described and prove themselves to participate in SLE pathogenesis. For instance, double negative T lymphocytes, expressing αβ T cells receptor, but lacking CD4, CD8, and Natural Killer surface markers, are recently described to proliferate and increase in SLE patients, probably following impaired tolerance of apoptotic cells, leading to IL-7 overproduction [49,50]. Furthermore, intracellular molecules, such as T-bet and CD11c, markers of age-associated B cells (ABC), are increased in SLE patients, but also following viral infections or vaccinations. Clinical importance is based on their sex differences, including population and function, which may explain differences in clinical presentation and outcome of SLE between males and females. Most interestingly, however, ABCs show similarities to advanced differentiated B lymphocytes, as they have been defined based on their IgD-CD27− phenotype [51]. This possible correlation between intra- and extra-cellular surface molecules, in terms of ageing and inflammation activity and chronicity, needs to be further investigated, with up-to-date findings predicting breakthrough results.

Despite the extensive evaluation of a remarkable number of lymphocyte markers and the definition of a whole cohort of certain subpopulations, our study has some limitations. The main limitation of the study is the unbalanced populations, in terms of gender, as the vast majority of SLE patients were females. Additionally, all estimated parameters are highly influenced by age, and no data are available if the age has the same impact in healthy control individuals and in chronic inflammatory conditions. We tried to rule the confounding effects of those parameters by performing additional statistics and describing relevant models; however, even our results still support the significant role of ageing on the top of the disease, in activation and senescence. It would be very interesting to assess the different effects of ageing in different chronic inflammatory or chronic autoimmune diseases.

In conclusion, in the present compared analysis of two different forms of chronic inflammation, we describe completely divergent effects, in terms of immunosenescent process. B lymphocyte compartment is clearly affected in the presence of SLE, with a clear shift towards senescent phenotype, something which is not evident in ESKD-HD patients. Instead, T lymphocyte subpopulations are affected in a completely different way, consisted of an increased proportion of central memory CD4 and CD8 cells in both conditions, yet reduced senescent phenotype in SLE patients and reduced naïve phenotype in ESKD-HD. It seems that in SLE, the senescent process affects B lymphocytes, while in ESKD-HD, it affects CD4 and CD8 lymphocytes. Our study is the first one to compare the senescent phenomenon in different inflammatory diseases and demonstrated that the effect of SLE on T lymphocytes seems to be reversible during remission, something which is not evident on the B cell compartment. On the other hand, the deleterious effect of uremia has more persistent and detrimental effects on the immune phenotype.

## 4. Materials and Methods

### 4.1. Patients

The study population consisted of 30 SLE, 53 ESKD patients on hemodialysis (ESKD-HD) and 31 healthy adult volunteers matched for age, sex, and race who served as healthy controls (HC). All participants were Greek, Caucasians, aged 18–67 years, and all were informed and signed the consent form before participating in the study.

Inclusion criteria: Regarding SLE patients, diagnosis of SLE should be based on the American Rheumatology Association (ARA) criteria, the patients should be stable with no flare up of lupus for at least 2 years, and their treatment for SLE, at time of evaluation, could include prednisolone, mycophenolate mofetil, calcineurin inhibitors, and hydroxychloroquine. Correspondingly, ESKD-HD patients should be on regular hemodialysis treatment for at least 2 years, and during this period being dialyzed under stable conditions, three times per week, achieving a KT/V > 1.5. Adequate treatment for anemia, hypertension, and hyperparathyroidism should be provided.

Exclusion criteria: Patients were excluded from the study in case of malignancy, diabetes mellitus, or hematological disorder. Patients with ESKD due to systemic disease, for instance rheumatoid arthritis, vasculitis, systemic lupus erythematosus, or diabetes mellitus were excluded. Patients with a recent, less than 6 months, history of infection or use of monoclonal antibodies or cyclophosphamide the last 2 years were also excluded from the study.

### 4.2. Schedule of the Study

This is a cross sectional study, including SLE patients followed in the joint Nephrology and Rheumatology outpatients clinic and patients undergoing hemodialysis in the Department of Nephrology in Hippokration Hospital. Information about clinical data, comorbid conditions, previous and current medication, and duration of the disease were retrieved from patients’ records. Activity of SLE was estimated based on the SLEDAI score, and laboratory findings, such as hematological, biochemical, and immunological profile, were recorded at time of evaluation.

### 4.3. Flow Cytometry

Heparinized venous blood from SLE patients, ESKD patients, and healthy control individuals was collected and proceeded for the evaluation of total lymphocytes, proportions and counts of B and T lymphocytes, CD4 and CD8 subtypes, and consequently expression of different surface molecules, determining divergent subpopulations. The expression of IgD and CD27 on B lymphocytes and of CD45RA, CCR7, CD28, CD31, CD57, on both CD4 and CD8 T lymphocytes was assessed using a cytometer (Navios Flow Cytometer, Beckman Coulter, Brea, CA, USA).

#### 4.3.1. Flow Cytometry Reagents

The following monoclonal antibodies were used to characterize the phenotypes of B lymphocytes: CD45 PC7, CD19 PC5, IgD FIC, and CD27 ECD and those of T lymphocytes: CD45 PC7, CD3 FITC, CD4 Pacific Blue, CD8 PC5, CD45RA APC, CCR7 PE, CD28 ECD, CD57 FITC, CD31 ECD. Three different tubes were used for the assessment of T cell and one tube for B cell markers, and the subpopulations defined based on combination as has been described before [12].

Gating strategies for B and T lymphocyte subpopulations are described on the supplement file, Supplement Figures S1 and S2.

#### 4.3.2. Lymphocyte Subpopulations

B lymphocyte senescent status was based on the expression of CD27 and IgD surface molecules, and subpopulations described were: naïve (CD19+IgD+CD27−), non-switched and switched memory (CD19+IgD+CD27+ and CD19+IgD+CD27−, respectively), and advanced differentiated or senescent (CD19+IgD-CD27−) cells.

Certain T lymphocyte subpopulations were categorized as (i) early differentiated cells, which included Recent Thymic Emigrants (RTE), defined as CD4+CD31+ and CD8+CD31+, and naïve, defined as CD4+CD45RA+CCR7+ and CD8+CD45RA+CCR7+ (ii) Memory cells, including Central (CM), CD4+CD45RA-CCR7+ and CD8+CD45RA-CCR7+, and Effector memory (EM), CD4+CD45RA-CCR7- and CD8+CD45RA-CCR7-, and finally (iii) Senescent/Advanced differentiated cells, including EMRA, that is CD4+CD45RA+CCR7− and CD8+CD45RA+CCR7−, but also CD28− and CD57+ lymphocytes (CD4+CD28−, CD4+CD45RA+CD28−, CD4+CD28-CD57−, CD4+CD28-CD57+, CD4+CD28+CD57+, CD4+CD45-CD57+, CD4+CD45+CD57+, CD8+CD28−, CD8+CD45RA+CD28−, CD8+CD28-CD57−, CD8+CD28-CD57+, CD8+CD28+CD57+, CD8+CD45-CD57+, CD8+CD45+CD57+) based on the combined expression of the senescent-related markers. All cell types and subtypes were defined based on certain surface molecules.

### 4.4. Statistics

Statistical analysis was performed using the Statistical Package for Social Sciences (SPSS Inc., Chicago, IL, USA) version 23.0 for Windows. Values of *p* < 0.05 (two-tailed) were considered as statistically significant. All continues variables were initially tested for normality of distribution, by using the Shapiro–Wilk and/or the Kolmogorov–Smirnov test.

Data from normally distributed variables were expressed as mean ± standard deviation, and Student’s t test for independent samples was performed to compare differences between two groups. Non–parametric variables were expressed as medians and range, or interquartile range, and differences between three groups were estimated by using the Kruskal–Wallis H test, followed by a post hoc analysis, with Dunn test, to compare differences within paired groups. Type II ANOVA was performed to estimate the effect of different disease types, namely SLE and ESKD-HD, on each lymphocyte subpopulation, corrected for age and gender. Therefore, a separate model was created, by using type II ANOVA, where the effect of disease types, as fixed factor, corrected for age and gender (covariate factors), resulting in the following model: immune cell population (cells/μL) ~ group + age + gender). The results of this model were compared to those from the Kruskal–Wallis H test, in order to estimate the specific effect of two covariate factors, age and gender, on the differences in lymphocyte counts between different diseases. In a similar way, type III ANOVA was subsequently performed, leading to a model which included the interaction between separate variables, that is: immune cell population (cells/μL) ~ group + age + gender + group × age + group × gender + group × age × gender.

Mann–Whitney U test was performed to estimate differences between two groups, such as duration of disease, between SLE and ESKD-HD patients, and Chi-square test to estimate sex differences between the three groups.

## Figures and Tables

**Table 1:** Demographic and laboratory data of patients (SLE and SKD-HD) and healthy controls.

	SLE	ESKD-HD	HC	*p* *	*p* *	*p* *	*p* *
n	30	53	31	KW Test	SLE vs. HC	HD vs. HC	SLE vs. HD
Female (%)	29 (96.7)	19 (35.8)	17 (54.8)	<0.0001	-	-	-
Age (yrs)	43 ± 14	47 ± 14	49 ± 13	NS	NS	NS	NS
Time since diagnosis (mo)	84 (45–125)	67 (28–84)	-	-	-	-	NS
Laboratory results							
WCC	7200 (3350)	7100 (1950)	6400 (1800)	NS	-	-	-
Neutrophils (%)	69.6 (20.7)	63.3 (9.6)	58.1 (10.35)	<0.0001	<0.0001	0.004	NS
Neutrophils	4600 (3500)	4550 (1675)	3500 (1200)	0.007	0.03	0.009	NS
Lymphocytes (%)	23.1 (16.1)	19.8 (8.9)	25.6 (9)	0.001	0.03	<0.0001	NS
Lymphocytes	1400 (900)	1500 (500)	2100 (900)	0.001	0.005	0.001	NS
NLR	3 (4)	2.8 (1.37)	1.8 (0.85)	<0.0001	<0.0001	<0.0001	NS

* Kruskal–Wallis test and Dunn test.

**Table 2:** Differences in B lymphocytes and subpopulations in HC, SLE and HD patients.

	SLE	ESKD-HD	HC	*p* *	*p* *	*p* *	*p* *
n	30	53	31	KW Test	SLE vs. HC	ESKD-HD vs. HC	SLE vs. ESKD-HD
CD19 **	75.4 (14.4–520.8)	97 (32–341)	214 (84–576)	<0.001	<0.001	<0.001	NS
IgD+CD27−	37.71 (0.26–434.84)	73 (2–303)	117 (5–364)	<0.001	<0.001	<0.001	NS
IgD+CD27+	5.12 (0.13–17.55)	5 (0–31)	23 (2–700)	<0.001	<0.001	<0.001	NS
IgD-CD27+	18.58 (0.47–89.58)	13 (1–75)	38 (11–258)	<0.001	0.001	<0.001	NS
IgD-CD27−	10.84 (0.93–122.91)	8 (1–132)	21 (3–202)	<0.001	0.007	<0.001	NS

* Kruskal–Wallis test and Dunn test, ** cells/Μl.

**Table 3:** Differences in early, memory, and late differentiated CD4 Lymphocytes and subpopulations in SLE and ESKD-HD patients and HC.

	SLE	ESKD-HD	HC	*p* *	*p* *	*p* *	*p* *
n	30	53	31	KW Test	SLE vs. HC	ESKD−HD vs. HC	SLE vs. ESKD−HD
CD4 **	651.2 (71.1–1478.2)	713 (234–1509)	986 (344–1591)	<0.001	0.004	0.001	NS
Early differentiated cells **							
CD4+CD31+	216.38 (16.3–904.7)	131 (27–560)	250 (69–967)	0.014	NS	0.01	NS
CD45RA+CCR7+	298.2 (21.3–1049.5)	242 (47–854)	359 (28–1357)	NS	NS	0.022	NS
CD4CD45RA+CD28+	267.97 (20.62–1030.31)	200 (7–768)	388 (139–1402)	0.001	NS	<0.001	NS
CD4CD28+CD57−	610.7 (54.68–1461.94)	631 (201–1346)	958 (332–1569)	<0.001	0.004	<0.001	NS
CD4CD45RA+CD57−	254.03 (21.05–1077.61)	258 (27–916)	401 (160–1373)	0.005	0.035	0.005	NS
CD4CD45RA-CD57−	290.67 (38.96–884.43)	375 (133–972)	539 (173–991)	<0.001	<0.001	0.01	NS
Memmory cells **							NS
CD45RA-CCR7+	402.35 (38.7–972.4)	384 (80–1015)	563 (40–1001)	NS	0.046	NS	NS
CD45RA-CCR7−	1.62 (0–73.49)	5 (0–109)	11 (0–590)	0.002	0.002	NS	0.01
Senescent/Advanced differentiated cells **							
CD45RA+CCR7−	7.29 (0–180.62)	14 (0–150)	23 (0–487)	0.031	0.027	NS	NS
CD4CD28−	20.12 (1.27–139.06)	42 (3–851)	38 (3–299)	0.02	NS	NS	0.01
CD4CD28-CD57−	11.82 (0–65.26)	20 (2–175)	13 (2–119)	NS	NS	NS	0.016
CD4CD28-CD57+	9.90 (0.46–73.8)	17 (0–783)	23 (0–274)	NS	NS	NS	0.041

* Kruskal–Wallis test and Dunn test, ** cells/μL.

**Table 4:** Differences in in early, memory, and late differentiated CD8 Lymphocytes and subpopulations between HC, SLE, and HD patients.

	SLE	ESKD-HD	HC	*p* *	*p* *	*p* *	*p* *
n	30	53	31	KW Test	SLE vs. HC	ESKD-HD vs. HC	SLE vs. ESKD-HD
CD8 **	414.8 (60.6–2017.8)	354 (121–1225)	454.5 (154–1310)	NS	NS	0.04	NS
Early differentiated cells **							
CD8+CD31+	88.19 (8.2–1047)	91 (4–576)	187.5 (8–541)	NS	NS	0.03 **	NS
CD8CD45RA+CD28+	113.56 (1.81–753.7)	86 (0–748)	212.5 (7–1257)	NS	NS	0.025 **	NS
CD8CD28+CD57−	249.45 (5.49–1362)	169 (46–569)	298 (95–646)	0.003	NS	<0.001	NS
CD8CD45RA+CD57−	63.65 (3.83–889.8)	69 (8–450)	133 (8–552)	NS	NS	0.039	NS
CD8CD45RA-CD57−	194.52 (1.8–945.1)	154 (6–936)	179 (28–555)	NS	NS	NS	NS
Memory cells **							
CD8CD45RA-CCR7+	171.52 (2.5–1417)	127 (0–1104)	123 (1–941)	NS	NS	NS	NS
CD8CD45RA-CCR7−	13.94 (0.59–92.37)	13 (0–136)	25 (0–355)	NS	NS	NS	NS
Senescent/Advanced differentiated cells **							
CD8CD45RA+CCR7-	11.13 (0–279.6)	20 (0–242)	49.5 (0–534)	0.043	0.04	NS	NS
CD8CD28−	87.83 (4.56–1361.2)	139 (18–839)	135 (36–633)	NS	NS	NS	NS
CD8CD28-CD57−	46.49 (0.33–796.1)	62 (9–364)	46 (7–332)	NS	NS	NS	NS
CD8CD28-CD57+	53.17 (0.83–571.04)	79 (1–746)	71 (0–470)	NS	NS	NS	NS
CD8CD45RA+CD28−	52.27 (2.13–263.6)	120 (6–716)	197.5 (9–783)	<0.001	<0.001	NS	0.005
CD8CD45+CD57+	10.05 (0.45–141)	26 (0–388)	20 (0–333)	0.021	0.03	NS	0.047

* Kruskal–Wallis test and Dunn test, ** cells/μL.

## Data Availability

Not applicable.

## Supplementary Materials

The following supporting information can be downloaded at: https://www.mdpi.com/article/10.3390/ijms232314688/s1.
