# Supplementary material for: Different Types of Chronic Inflammation Engender Distinctive Immunosenescent Profiles in Affected Patients
Source: Int J Mol Sci. 2022 Nov 24;23(23):14688. doi: 10.3390/ijms232314688 (PMC9735546; doi:10.3390/ijms232314688)
Supplement: Supplementary file 1 [file ijms-23-14688-s001.zip › ijms-1979896-supplementary.pdf]

Suppl Table S1. Current immunosuppressive treatment in SLE patients

	SLE patients	
Current Immunosuppresive treatment	**N(%)**	**Time (mo)**
Hydroxychloroquin	30(100)	84(45-125)
Steroids	24(80)	78(45-92)
MMF	20(67)	53(37-65)
CNIs	12(40)	42(28-56)

Suppl Table S2. B Lymphocytes and subpopulations in SLE and ESKD-HD female patients and female HC

	**SLE**	**ESKD-HD**	**HC**	**p**	**p**		**p**
**n**	**29**	**19**	**17**	**KW test***	**SLE vs. HC**	**ESKD-HD vs. HC**	**SLE vs. ESKD-HD**
CD19%	5.8(2.1-28.6)	6.9(4.6-21)	10.2(5.4-24)	NS			
CD19 cells/μL	72.8(14.4-520.8)	97(46-279)	186(84-502)	0.003	0.002	NS	NS
IgD+CD27-%	51.5 (0.4-94)	69.9(45-90)	55(27.8-81.5)	0.04	NS	NS	0.02
IgD+CD27- cells/μL	37.07(0.26-435)	79(27-203)	105(46-317)	0.01	0.01	NS	NS
IgD+ CD27+%	5(0.2-22.5)	6.5(1.3-30.9)	10.4(3-44)	0.028	0.02	NS	NS
IgD+CD27+ cells/μL	5.1(0.13-17.55)	8(2-31)	17(5-147)	<0.001	<0.001	0.01	NS
IgD-CD27+%	19.2(2.2-78)	16(3.1-28.4)	17.2(7.7-39.7)	NS			
IgD-CD27+ cells/μL	18.44(0.47-89.58)	16(4-66)	28(11-148)	0.006	0.03	0.006	NS
IgD-CD27-%	11.75(2.3-74.2)	6.4(3-19.8)	91(4.7-26.5)	0.006	NS	NS	0.004
IgD-CD27- cells/μL	10.64(0.93-122.91)	7(3-35)	19(7-62)	0.003	NS	0.002	NS

*Kruskal-Wallis test and Dunn test was performed to estimate differences between two groups, SLE vs. HC, ESKD-HD vs. HC and SLE vs. ESKD-HD

Suppl Table S3. CD4 Lymphocytes and subpopulations in SLE and ESKD-HD female patients and female HC

	**SLE**	**ESKD-HD**	**HC**	**p**	**p**	**p**	**p**
**n**	**29**	**19**	**17**	**KW test***	**SLE vs. HC**	**ESKD-HD vs. HC**	**SLE vs. ESKD-HD**
CD4%	49.6(21.6-71.8)	49.8(32.1-63.3)	53.6(36.1-70.4)	NS			
CD4 cells/μL	637(71.1-1478)	679(371-1329)	981(442-1591)	0.014	0.018	0.047	NS
Early differentiated cells							
CD4CD28+CD57- cells/μL	599.1(55-1462)	603(367-1319)	956(425-1569)	0.019	0.022	0.024	NS
CD4CD45-CD57- cells/μL	287.18 (39-884)	393(160-738)	561(208-991)	0.002	0.001	NS	NS
Memory cells							
CD45RA-CCR7- cells/μL	1.68(0-73.5)	3(0-86)	11(1-590)	0.01	0.008	NS	NS
Senescent/Advanced differentiated cells							

CD45RA+CCR7-cells/μL	6.93(0-181)	14(1-95)	23(1-487)	0.034	0.029	NS	NS

*Kruskal-Wallis test and Dunn test was performed to estimate differences between two groups, SLE vs. HC, ESKD-HD vs. HC and SLE vs. ESKD-HD,

Suppl Table S4. CD8 Lymphocytes and subpopulations in SLE and ESKD-HD female patients and female HC

	**SLE**	**ESKD-HD**	**HC**	**p**	**p**	**p**	**P**
**n**	**29**	**19**	**17**	**KW test***	**SLE vs. HC**	**ESKD-HD vs. HC**	**SLE vs ESKD-HD**
CD8%	29.3(12.8-58.5)	21.2 (11.1-33.6)	21.7 (10.8-52.4)	0.023	NS	NS	0.034
CD45RA+CCR7-cells/μL	10.84(0-279.6)	36 (1-205)	133 (0-534)	0.041	0.039	NS	NS
CD8CD45RA+CD28- %	8.6(1.3-35.7)	13.4 (1.5-75.1)	20.5 (2-59)	0.029	0.027	NS	NS
CD8CD45RA+CD28-cells/μL	49.5(2.13-263.6)	102 (6-716)	262 (9-783)	0.001	0.001	NS	NS

*Kruskal-Wallis test and Dunn test was performed to estimate differences between two groups, SLE vs. HC, ESKD-HD vs. HC and SLE vs. ESKD-HD

Suppl Table S5. Heatmap/table showing the correlation of Age and Sex in the peripheral count of B lymphocytes and their subpopulations

	**B lymphocytes - Correlation Coefficient**					
	**Age**	**CD19**	**CD19 IgD+ CD27-**	**CD19 IgD+ CD27+**	**CD19 IgD- CD27+**	**CD19 IgD- CD27-**
**Age**	1	-0.2610	-0.2910	-0.0600	-0.0790	-0.2210
**CD19**	-0.2610	1	0.9150	0.6810	0.6880	0.6910
**CD19 IgD+ CD27-**	-0.2910	0.9150	1	0.5920	0.4580	0.4650
**CD19 IgD+ CD27+**	-0.0600	0.6810	0.5920	1	0.7060	0.4010
**CD19 IgD- CD27+**	-0.0790	0.6880	0.4580	0.7060	1	0.6720
**CD19 IgD- CD27-**	-0.2210	0.6910	0.4650	04010	0.6720	1
	**B lymphocytes - Significance (2-tailed)**					
	**Age**	**CD19**	**CD19 IgD+ CD27-**	**CD19 IgD+ CD27+**	**CD19 IgD- CD27+**	**CD19 IgD- CD27-**
**Age**	.	0.0080	0.0030	0.5440	0.4270	0.0250
**CD19**	0.0080	.	0.0000	0.0000	0.0000	0.0000
**CD19 IgD+ CD27-**	0.0030	0.0000	.	0.0000	0.0000	0.0000
**CD19 IgD+ CD27+**	0.5440	0.0000	0.0000	.	0.0000	0.0000
**CD19 IgD- CD27+**	0.4270	0.0000	0.0000	0.0000	.	0.0000
**CD19 IgD- CD27-**	0.0250	0.0000	0.0000	0.0000	0.0000	.

Suppl Table S6. Heatmap/table showing the correlation of Age and Sex in the peripheral count of T lymphocytes and their subpopulations

	T lymphocytes - Correlation Coefficient									
	Age	CD4	CD4CD31+	CD4 CD45RA+CCR7+	CD4 CD45RA-CCR7+	CD4 CD45RA-CCR7-	CD4CD28-	CD4 CD45+CD57+	CD4 CD28-CD57+	CD8 D45RA+CCR7-
**Age**	1	-0.229	-0.415	-0.232	-0.092	-0,11	-0.044	0.059	0.131	-0.088
**CD4**	-0.229	1	0.685	0.742	0.73	0.25	0.166	0.261	0.126	0.297
**CD4 CD31+**	-0.415	0.685	1	0.851	0,292	0.051	0.086	0.226	-0.011	0.22
**CD4 CD45RA+CCR7+**	-0.232	0.742	0.851	1	0.445	-0.022	0.095	0.32	0.094	0.212
**CD4 CD45RA-CCR7+**	-0.092	0.73	0.292	0.445	1	0.14	0.094	0.245	0.139	0,086
**CD4CD45RA-CCR7-**	-0.11	0.25	0.051	-0.022	0.14	1	0.31	0.083	0.194	0.513
**CD4CD28-**	-0.044	0.166	0.086	0.095	0.094	0.1	1	0.673	0.863	0.322
**CD4CD45+CD57+**	0.059	0.261	0.226	0.32	0.245	0.083	0.673	1	0.798	0.333
**CD4CD28-CD57+**	0.131	0.126	-0.011	0.094	0.139	0.194	0.863	0.798	1	0.315
**CD8D45RA+CCR7-**	-0.088	0.297	0.22	0.212	0.086	0.513	0.322	0.333	0.315	1
	**T lymphocytes - Significance (2-tailed)**									
	**Age**	**CD4**	**CD4CD31+**	**CD4CD45RA+CCR7+**	**CD4CD45RA-CCR7+**	**CD4CD45RA-CCR7-**	**CD4CD28-**	**CD4 CD45+CD57+**	**CD4 CD28-CD57+**	**CD8 D45RA+CCR7-**
**Age**	.	0.0190	0.0000	0.0180	0.3530	0.2700	0.6570	0.5570	0.1840	0.3720
**CD4**	0.0190	.	0.0000	0.0000	0.0000	0.0080	0.0780	0.0050	0.1810	0.0010
**CD4 CD31+**	0.0000	0.0000	.	0.0000	0.0020	0.5920	0.3640	0.0160	0.9080	0.0190
**CD4 CD45RA+CCR7+**	0.0180	0.0000	0.0000	.	0.0000	0.8130	0.3150	0.0010	0.3230	0.0240
**CD4 CD45RA-CCR7+**	0.3530	0.0000	0.0020	0.0000	.	0.1390	0.3230	0.0090	0.1430	0.3680
**CD4CD45RA-CCR7-**	0.2700	0.0080	0.5920	0.8130	0.1390	.	0.0010	0.3830	0.0390	0.0000
**CD4CD28-**	0.6570	0.0780	0.3640	0.3150	0.3230	0.0010	.	0.0000	0.0000	0.0000
**CD4CD45+CD57+**	0.5570	0.0050	0.0160	0.0010	0.0090	0.3830	0.0000	.	0.0000	0.0000
**CD4CD28-CD57+**	0.1840	0.1810	0.9080	0.3230	0.1430	0.0390	0.0000	0.0000	.	0.0010
**CD8D45RA+CCR7-**	0.3720	0.0010	0.0190	0.0240	0.3680	0.0000	0.0000	0.0000	0.0010	.

Suppl Table S7. Adjustment of Age and Sex in the expression of CD19, CD4 and CD8 lymphocytes and their subpopulations

	Type II ANOVA			Type III ANOVA		
cells/µL	Group^	Age	Sex	Group*Age	Group*Sex	Group*Age*Sex
**CD19**	**<0.001**	**0.022**	0.703	**0.013**	0.232	0.300
CD19IgD+CD27-	**0.003**	**0.002**	0.921	0.054	0.067	0.217
CD19IgD+CD27+	**0.021**	0.599	0.413	0.116	0,976	0,710
CD19IgD-CD27+	**<0.001**	0.813	0.790	**0.003**	0.299	0.100
CD19IgD-CD27-	**0.023**	0.586	0.169	0.313	0.753	0.894
**CD4**	**<0.001**	**0.027**	0.772	0.685	0.937	0.947
Early differentiated cells	-					
CD4CD31+	**0.003**	**<0.001**	0.165	0.117	0.923	0.621
CD4CD45RA+CCR7+*	**0.026**	0.105	0.373	0.343	0.898	0.735
CD4CD45RA+CD28+	**<0.001**	**0.008**	0.294	0.192	0.822	0.614
CD4CD28+CD57-	**<0.001**	**0.021**	0.479	0.439	0.964	0.903
CD4CD45RA+CD57-	**0.005**	**0.015**	0.547	0.179	0.746	0.778
CD4CD45RA-CD57-	**<0.001**	0.170	0.927	0.661	0.689	0.607
Memory cells	-					
CD4CD45RA-CCR7+	0.122	0.361	0.475	0.798	0.521	0.583
CD4CD45RA-CCR7- *	0.076	0.245	0.657	0.997	0.112	0.502
Senescent/Advanced differentiated cells	-					
CD4CD45RA+CCR7-	**0.049**	0.089	0.898	0.863	0.195	0.529
CD4CD28-*	0.764	0.321	0.110	0.848	0.677	0.785
CD4CD28-CD57-	0.598	0.06	0.139	0.466	0.299	0.699
CD4CD28-CD57+	0.883	0.087	0.184	0.529	0.706	0.786
**CD8 ***	**0.046**	0.558	0.258	0.892	0.222	0.821
Early differentiated cells	-					
CD8+CD31+	0.096	0.813	0.276	0.972	0.271	0.692
CD8CD45RA+CD28+ *	**0.01**	**0.03**	0.184	0.780	0.085	0.501
CD8CD28+CD57-	**0.001**	**0.023**	0.807	0.199	0.466	0.857
CD8CD45RA+CD57- *	**0.036**	0.133	0.887	0.876	0.128	0.417
CD8CD45RA-CD57-	0.366	0.612	0.329	0.600	0.105	0.632
Memory cells	-					
CD8CD45RA-CCR7+	0.231	0.215	0.120	0.929	0.067	0.344
CD8CD45RA-CCR7- *	**0.025**	0.589	0.617	0.863	0.942	0.961
Senescent/Advanced differentiated cells	-					

CD8CD45RA+CCR7-	**0.008**	0.169	0.180	0.932	**0.003**	0.194
CD8CD28-	0.690	**0.033**	0.125	0.891	0.201	0.701
CD8CD28-CD57+	0.529	**0.006**	0.102	0.648	0.739	0.804
CD8CD45RA+CD28-	**0.001**	0.333	0.350	0.881	0.506	0.926
CD8CD45RA+CD57+ *	0.072	**0.018**	0.666	0.476	0.989	0.983

*Lymphocyte subpopulations which showed different significance after corrected for age and gender, compared to results from Kruskal Wallis H test, described on tables 2, 3 and 4

^Group: SLE, ESKD-HD and HC

Legends for Supplement Figures

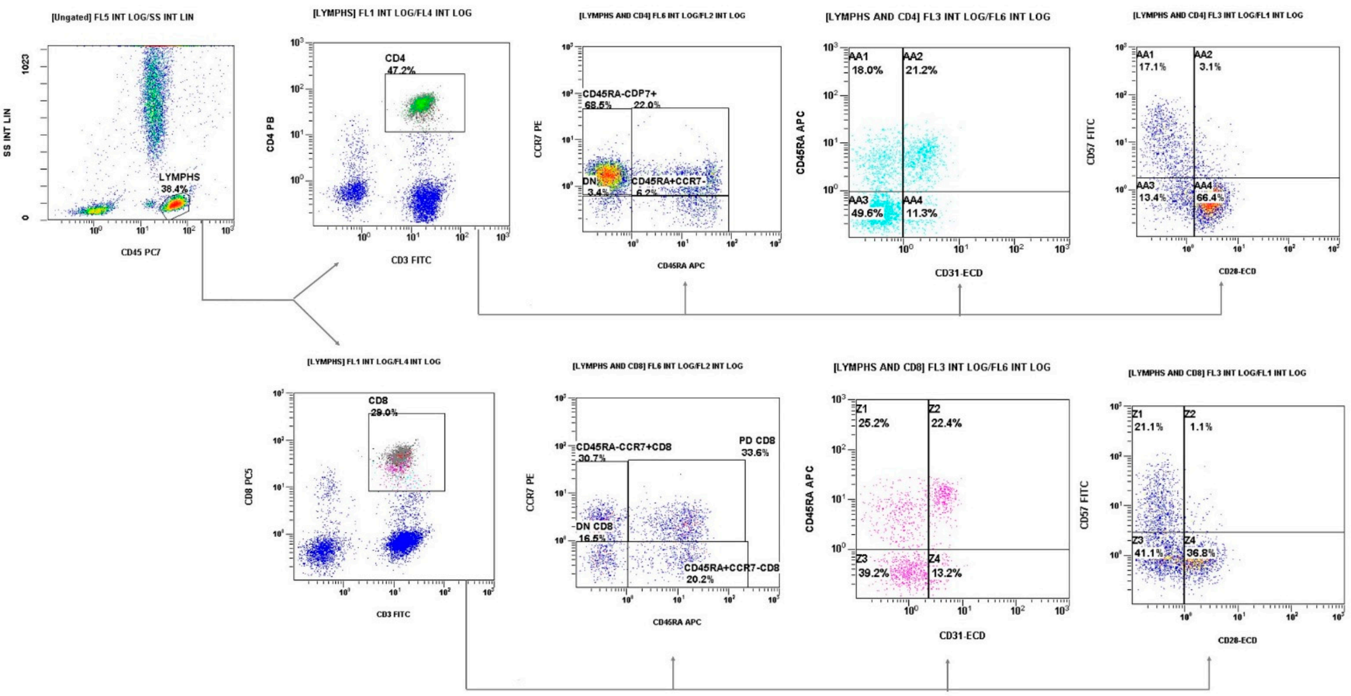

Suppl Figure S1. Description of gating process for T lymphocytes. Gating of Lymphocytes, CD3+CD8+ cells, CD8+CD45RA+CCR7+ cells, CD8CD28+CD57+ cells, CD8CD45RA+CD31+ cells

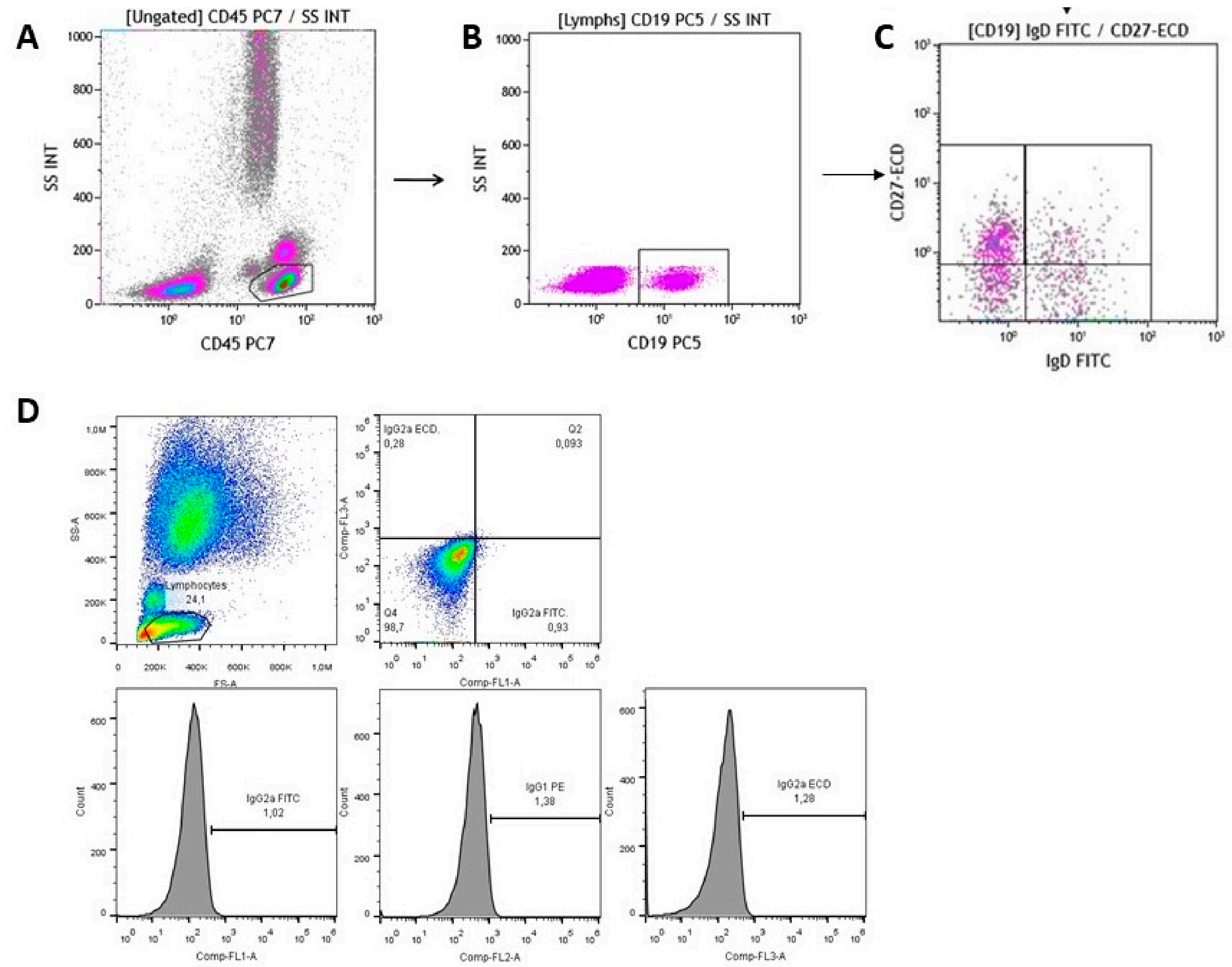

Suppl Figure S2. Description of gating process for B lymphocytes. Gating of lymphocytes (A), CD19+ cells (B) CD19IgD_CD27+ cells (C) and isotype control for IgD FITC and CD27 ECD (D)

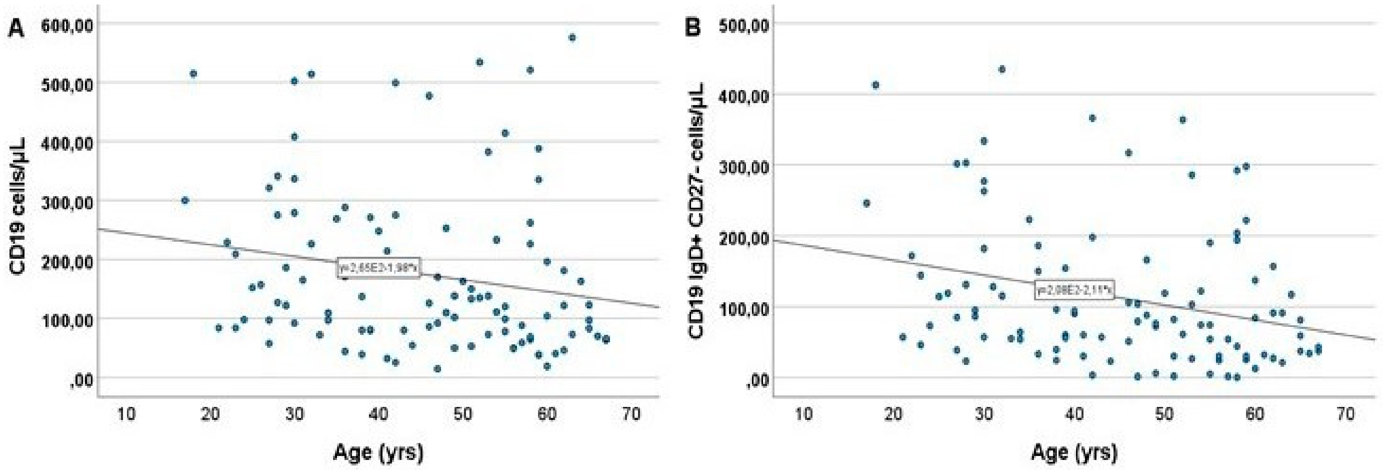

Suppl Figure S3. Correlation of Age with CD19 (A) and CD19IgD+CD27- cells (B) in the whole cohort of participants

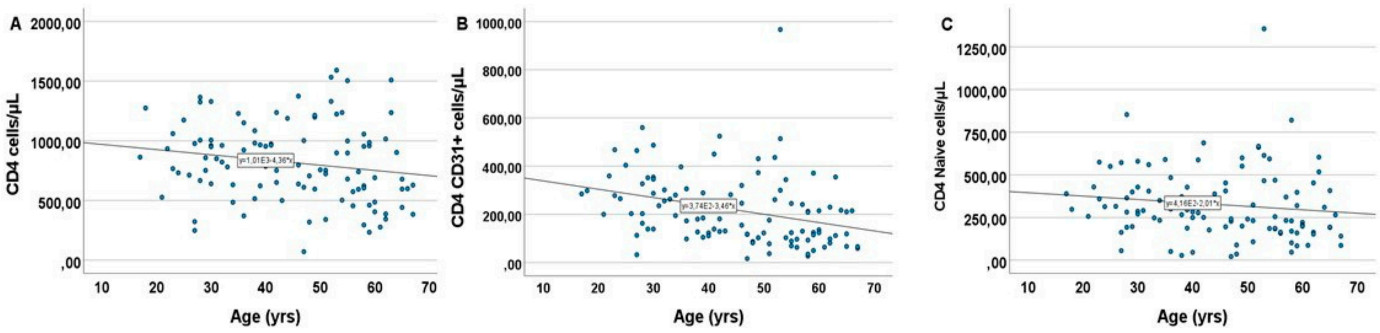

Suppl Figure S4. Correlation of Age with CD4 (A) and CD4CD31+ (B) and naïve CD4 cells (C) in the whole cohort of participants

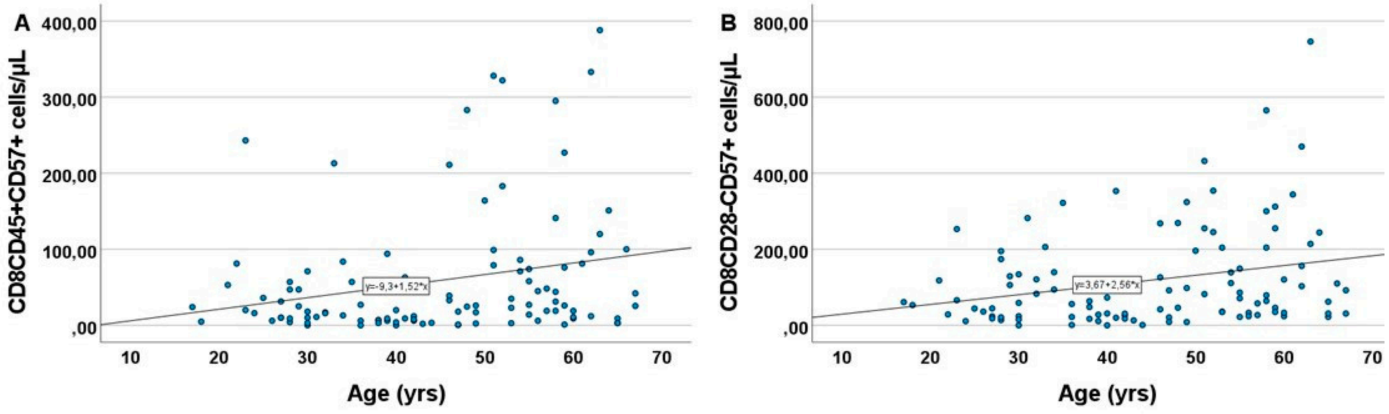

Suppl Figure S5. Correlation of Age with CD8CD45RA+CD57+ (A) and CD8CD28-CD57+ (B) cells n the whole cohort of participants
